# Risk Factors for Criminal Recidivism Among Persons With Serious Psychiatric Diagnoses: Disentangling What Matters for Whom

**DOI:** 10.3389/fpsyt.2021.778399

**Published:** 2021-12-16

**Authors:** Leah A. Jacobs, Alex Fixler, Travis Labrum, Ashley Givens, Christina Newhill

**Affiliations:** ^1^School of Social Work, University of Pittsburgh, Pittsburgh, PA, United States; ^2^School of Social Work, College of Human Environmental Sciences, University of Missouri, Columbia, MO, United States

**Keywords:** criminal recidivism, serious mental disorders, co-occurring disorders, substance use disorder, risk factors

## Abstract

Reducing criminal legal system involvement requires an understanding of the factors that promote repeat offending (i. e., recidivism), and the dissemination of relevant interventions to those most likely to benefit. A growing body of research has established common recidivism risk factors for persons with serious psychiatric disorder diagnoses. However, research to date has not examined the degree to which these risks apply to those with serious psychiatric disorders with and without co-occurring substance use disorders. To clarify what risk and need factors are greatest and for whom, this cross-sectional study drew from an original dataset containing data on 14 social and economic, psychological, and criminal risk areas for a cohort of people on probation (*n* = 4,809). Linear regression models indicated that, compared to those without a serious psychiatric disorder, people on probation with a serious psychiatric disorder are at greater risk in a minority of areas and those areas are mostly social and economic in nature. Meanwhile, those withco-occurring disorders are at relatively high risk across almost all areas. The results from this study suggest that justice involved persons with serious psychiatric disorders will benefit from interventions that increase social support and economic well-being and that interventions that broadly reduce risk among people with co-occurring serious psychiatric and substance use disorders will likely yield meaningful reductions in system involvement. Ultimately, understanding and intervening upon risk for recidivism among persons with serious psychiatric disorders requires differentiating between those with and without co-occurring substance use disorders.

## Introduction

The overrepresentation of people with serious psychiatric disorders (SPD) in criminal legal systems is of practical and ethical concern. Criminal legal systems typically lack the infrastructure to appropriately meet the needs of people with SPD, and persons with these disorders often struggle to safely and effectively navigate these systems [for a review, see Mulvey and Schubert (1)]. Further, for many people with SPD and especially for those with SPD and co-occurring substance use disorders (COD), system involvement begets future involvement (1, 2), making recidivism reduction a key goal for reducing overrepresentation. Identifying recidivism risk factors—i.e., factors that correlate with recidivism and precede recidivism in time (3)—that are relevant to persons with SPD and COD, is a first step toward achieving this goal.

A large body of research has established risk factors for criminal behavior in the general population (4). Derived from this research, Bonta and Andrews (5, 6) organize criminogenic risk factors into three categories—minor (family of origin, demographics, temperament, mental health, and neighborhood characteristics), moderate (education/employment, family/marital relationships, substance use, and antisocial recreational activities), and major (pro-criminal companions, attitudes and cognitions in support of criminal behavior, antisocial personality pattern, and history of criminal behavior). Factors in the moderate and major groups (i.e., “the central 8”) are consistent predictors of criminal behavior in the general population. Factors that are variable, or “dynamic” (e.g., employment, substance use, antisocial activities, companions, and attitudes), can theoretically be targeted to reduce future criminal behavior (5). Though assessment tools often confirm that the central 8 are correlated with criminal justice outcomes across various sub-populations (e.g., youth, indigenous people, people convicted of sex offenses), there is some variation in their relative importance across groups (6).

Given potential variation across groups, some researchers have assessed risk factors for system-involved persons with psychiatric disorders. Looking within samples of “mentally disordered offenders,” a meta-analysis (*k* = 126) of risk factors for criminal recidivism found that substance abuse was the strongest predictor of general recidivism, followed by procriminal attitudes and cognitions, and a criminal personality pattern (6). Meanwhile, clinical variables (e.g., diagnoses and hospitalization history) had relatively little effect on recidivism. The authors were unable to assess the role of antisocial peers or leisure/recreation, as these factors had been tested in too few studies. In a separate study, Skeem et al. (7) compared parolees with SPD (including those with psychotic, bipolar, and major depressive disorders) to those without SPD (*n* = 221), finding parolees with SPD had higher levels of risk across domains, with statistically significant differences in employment/education, family/marriage, procriminal attitudes, and antisocial personality patterns. Differences were not statistically significant for criminal history, leisure/recreation, companions, or alcohol/drugs. However, when assessing factors that maximally predict recidivism for those with and without SPD, Skeem et al. found that substance abuse and antisocial companions added predictive utility for the SPD group but not those without SPD. Together, these studies suggest that system-involved people with psychiatric disorders share recidivism risk factors with their relatively well counterparts, but also that they are relatively high in many of these shared factors [see also Morgan et al. (8) and Wilson et al. (9)] and may be at particularly increased risk of recidivism due to substance abuse and antisocial peers (7).

In our view, current research may mischaracterize risk among persons with SPD by neglecting the role of co-occurring substance use disorders (SUD) in shaping risk. An estimated 29% of male and 52% of female gaol inmates meet criteria for SUD (10, 11) and up to 75% of those with SPD have substance use problems (12, 13). The high prevalence of substance use problems among those with and without SPD, and their balance within study samples, may mask differences in criminogenic risk between those with SPD only and COD. Further, given substance use is a stable predictor of recidivism and persons with COD recidivate more often than those without (2), the high prevalence of SUD among those with psychiatric problems may inflate risk scores among persons with SPD. Ultimately, without accounting for the presence of co-occurring SUD, research to date may fail to identify differences in the constellation of risk factors experienced by those with and without psychiatric disorders, and may erroneously inflate risk among persons with SPD only.

Identifying relevant risk factors for recidivism for different groups has important implications for supervision decisions, delivering interventions to those most likely to benefit, and informing the substance of interventions. Though prior research has helped identify risk factors for justice-involved persons with SPD, the relevance of these risk factors when co-occurring SUD are taken into account remains unclear. This study addresses this gap by asking whether the distribution of recidivism risk factors varies across people without SPD, with SPD, and with COD. Based on prior research findings that indicate people with SPD are high in risk factors relative to those without SPD, substance use is a risk factor for recidivism, and recidivism rates are elevated among persons with COD, we hypothesize that risk factors will vary by diagnostic group, with those without SPD experiencing the least criminogenic risk, those with SPD experiencing greater risk than those without SPD, and those with COD experiencing the greatest risk. We test this hypothesis based on a sample of 4,809 people on probation and data on 14 risk domains from a popular, validated criminal risk assessment instrument. Results provide guidance for recidivism reduction.

## Method

This study is observational and cross-sectional. We utilize data on a cohort of people on probation in San Francisco, California. We further describe our sample, data, and analyses below.

### Sample

We obtained data on all people who began probation in San Francisco between September 2009 and August 2015 (*N* = 6,612). We excluded 1,800 people from the dataset due to missing or incomplete Correctional Offender Management Profiling for Alternative Sanctions (COMPAS) scores and an additional 3 people due to missing gender data, leaving 4,809 people in the dataset. Nearly 10% (*n* = 472) were diagnosed with either SPD only (*n* = 230) or COD (*n* = 242). See Table 1 for demographic and risk factor distribution.

**Table 1:** Demographic and subscale score distribution across diagnostic groups.

**Variable**	**(1) No SPD** **(*n* = 4,337, 90.19%)**	**(2) SPD(*n* = 472, 9.81%)**	**(3) SPD Only** **(*n* = 230, 4.78%)**	**(4) COD(*n* = 242, 5.03%)**	**(5) Total** **(*n =* 4,809)**
Age	35.54	39.06 [11.08]	38.87 [11.78]	38.38 [11.59]	35.89 [12.00]
Race					
Black	1913 (44.11)	195 (41.31)	97 (42.17)	98 (40.50)	2108 (43.83)
White	1031 (23.77)	193 (40.89)	92 (40.00)	101 (41.74)	1224 (25.46)
Another race/ethnicity	1179 (27.18)	76 (16.10)	34 (14.78)	42 (17.36)	1255 (26.10)
Unknown/Not reported	214 (4.93)	8 (1.69)	7 (3.04)	1 (0.004)	222 (4.62)
Gender					
Male	3768 (86.88)	390 (82.63)	198 (86.09)	192 (79.34)	4,158 (86.46)
Female	569 (13.12)	82 (17.37)	32 (13.91)	50 (20.66)	651 (13.54)
Recidivism risk factors					
Social environment	5.46 [2.87]	5.90 [2.86]	5.45 [2.80]	6.33 [2.85]	5.50 [2.87]
Criminal involvement	5.42 [2.90]	6.19 [2.55]	5.37 [2.57]	6.97 [2.27]	5.50 [2.87]
Hx of non-compliance	5.45 [2.87]	5.94 [2.84]	5.23 [2.72]	6.62 [2.79]	5.50 [2.87]
Substance abuse	5.40 [2.86]	6.40 [2.82]	5.37 [2.94]	7.39 [2.32]	5.50 [2.87]
Residential instability	5.34 [2.85]	6.96 [2.68]	6.61 [2.81]	7.29 [2.52]	5.50 [2.87]
Social isolation	5.35 [2.83]	6.86 [2.89]	6.68 [2.92]	7.02 [2.85]	5.50 [2.87]
Vocational/education	5.39 [2.88]	6.50 [2.61]	6.36 [2.71]	6.62 [2.51]	5.50 [2.87]
Criminal attitudes	5.44 [2.85]	6.08 [3.01]	5.90 [3.10]	6.24 [2.91]	5.50 [2.87]
Financial	5.47 [2.87]	5.79 [2.87]	5.56 [2.79]	6.00 [2.93]	5.50 [2.87]
Family criminality	5.51 [2.85]	5.43 [3.05]	5.25 [2.91]	5.61 [3.17]	5.50 [2.87]
Leisure and recreation	5.39 [2.85]	6.51 [2.85]	6.27 [2.83]	6.73 [2.86]	5.50 [2.87]
Criminal personality	5.43 [2.87]	6.17 [2.85]	5.77 [2.92]	6.55 [2.73]	5.50 [2.87]
Criminal associates/Peers	5.49 [2.85]	5.58 [3.06]	5.02 [3.09]	6.12 [2.93]	5.50 [2.87]
History of violence	5.42 [2.86]	6.20 [2.92]	5.97 [2.85]	6.42 [2.97]	5.50 [2.87]

*Means and standard deviations presented for continuous variables and counts and proportions presented for nominal variables. All recidivism risk factors are measured as deciles (i.e., where 1 point difference is equal to a 10% difference in rank; see Appendix for further detail on scales and the interpretation of decile scores). The categories SPD and No SPD include those both with and without an SUD, but the category SPD Only does not include individuals with an SUD*.

Comparing those included and excluded due to missing data, we found that there were significant race, gender, diagnosis, and offense severity differences; those included were more likely to be Black (43.83 vs. 22.46%) and less likely to be White [25.46 vs. 30.34%; *F*_(5, 6020)_ = 38.71, *p* < 0.001]; more likely to be male (86.46 vs. 77.54%; χ^2^ = 82.89, *p* < 0.001); more likely to have SPD (9.81 vs. 5.49%; χ^2^ = 30.53, *p* < 0.001) and COD (5.03 vs. 1.83%; χ^2^ = 32.93, *p* < 0.001); and more likely to have committed a felony offense (84 vs. 30%; χ^2^ = 1,751.80, *p* < 0.001). Given the prevalence of felony offenses, the caseload in the study site, and especially our sample, is relatively high in offense severity for a cohort of people on probation.

### Data and Measures

#### Data Sources

Diagnostic and demographic data were obtained from behavioral health service records and probation case records, respectively. Diagnoses were coded from an electronic health records system that tracks clinical information from all publicly funded providers in San Francisco (i.e., those who serve Medicaid/Medicare-eligible clients or receive funding *via* the County general fund). This system is not used by all providers and does not allow capturing all participants who have received related diagnoses (e.g., participants that have received relevant diagnoses by a provider utilizing private insurance may not be captured). However, we have likely captured the overwhelming majority of those with relevant, existing diagnoses. Public insurance is widely accessible in San Francisco, and many people on probation receive public services due to income constraints and employment difficulties. The data include records from providers that administer a variety of services, including residential, therapeutic, case management, medication management, crisis, inpatient, and court-ordered. Finally, the rates of service use in the present dataset are similar to those represented in other samples of people with SPD and SUD (14).

Recidivism risk factor scores were sourced from the COMPAS risk assessment, which includes self-report and criminal record data. COMPAS is a tool designed to evaluate recidivism risk factors among individuals involved in criminal legal systems. In addition to an overall risk score, COMPAS contains 15 base scales that measure risks in different domains [see Demarais et al. (15) and Appendix]. We selected 14 of these for analysis. We excluded current violence, as it was less a risk scale and more an indicator of the index offense, and because history of violence was substantively similar but more robust. Prior studies have indicated the overall validity of COMPAS in predicting recidivism (15, 16), including in the study site (17), and support the predictive validity and reliability of the majority of the base scales (16). We conducted separate reliability and validity tests, finding that the majority of scales were internally consistent (see below) and showed signs of construct validity (i.e., all correlation coefficients were positive and, for the most part, correlated in theoretically anticipated directions).

#### Measures

Predictor variables included diagnostic status and diagnostic group. Diagnostic status refers to the presence of a serious psychiatric disorder (0 = not present, 1 = present). In agreement with previous research, we defined the presence of SPD as having a documented diagnosis of a psychotic disorder, bipolar disorder, and/or major depression with psychotic features or classified as severe. We included and measured diagnostic status without differentiating those with and without substance use disorders in order to situate our results in the context of prior studies. However, our primary predictor of interest is diagnostic group, a nominal variable indicating a person has no SPD (reference category), SPD without a co-occurring SUD, or COD. We included all substance use disorders in our definition of SUD, including alcohol and drug, with the exception of nicotine use disorders. All diagnoses were documented by licensed clinicians, using the Diagnostic and Statistical Manual of Mental Disorders, 4th and 5th editions (18, 19).

Outcome variables were 14 recidivism risk factors, including criminal, social and economic, and psychological factors. For each risk factor, we converted the COMPAS raw score into deciles to permit comparison of scales and aid interpretation; each 1-point increase is equivalent to a 10% increase in ranking, and for most scales a score equal to or >6 is considered “moderate risk” and a score equal to or >8 is considered “high risk” (see Appendix for further detail). Criminal factors included criminal involvement (i.e., prior involvement in the criminal legal system); history of non-compliance (i.e., prior community supervision failure); and history of violence (i.e., violence in a person's legal history). Social and economic factors included social environment (i.e., crime, disorder, and victimization in a person's neighborhood and social groups); residential instability (i.e., the amount a person moves and lacks a residence); social isolation (i.e., lack of support in a person's social network); criminal associates (i.e., associating with people who use drugs, are involved with legal systems, or are members of a gang); family criminality (i.e., legal system involvement and substance abuse among family members); vocational/education (i.e., lack of and problematic work and education experience); and financial (i.e., poverty and financial stress). Psychological factors included criminal attitudes (i.e., beliefs that serve to rationalize illegal actions); criminal personality (i.e., personality traits associated with criminal actions); substance abuse (i.e., current and prior involvement in substance use and treatment), and leisure and recreation (i.e., feelings of boredom or distractibility). Internal consistency was acceptable or good for all scales, with the exception of history of violence (α = 0.63) and financial (α = 0.64; see Appendix).

Because the diagnostic groups differed demographically and risk can vary by demographic characteristics [see, e.g., Monahan et al. (3)], we included key demographic variables as controls. Gender was measured as a binary variable (1 = male, 0 = female). Race was measured as a nominal variable, including the categories of Black (reference category), White, Other, and Unknown/Not Reported. Age was measured in years from birth at probation start.

### Analysis

Two sets of analyses were conducted to assess whether diagnostic status or group predicted risk scores for the 14 risk areas. In the first set, we used 14 ordinary least squares regression (OLS) models to test whether diagnostic status, adjusting for demographics, was associated with each risk scale. In the second set, we used 14 OLS regression models to examine the relationship between diagnostic group and each risk scale, adjusting for demographics. We assessed and found no evidence of model assumption violation for any model. Finally, in *post-hoc* analyses, we assessed differences between persons with SPD and COD using Tukey pairwise comparison tests. For the first set of regressions, second set of regressions, and Tukey tests, we adjusted *p*-values for multiple comparisons using the Holm method. Analyses were conducted using R statistical computing software (20).

## Results

### Sample Description: Variation in Risk Factors by Diagnostic Status and Group

Table 1 presents descriptive data on risk factors in the sample (*n* = 4,809). Descriptive data illustrate variation in risk by diagnostic status and group (No SPD, SPD only, and COD). Compared to those without serious diagnoses (with and without SUD; column 1), those with SPD (with and without SUD; column 2) have greater risk scores across all domains, except family criminality. However, when comparing those without SPD (column 1) to those with SPD only (column 3), the distributions of risk for persons with SPD only are similar (criminal history, financial, social environment, and substance abuse), lower (criminal associates, family criminality, and history of non-compliance), or greater (criminal attitudes, criminal personality, history of violence, leisure, residential instability, support, and vocational/education). Meanwhile, compared to those without SPD (column 1) and with SPD only (column 3), those with COD (column 4) have higher risk scores across all domains. The average rank for COD group members exceeds or approaches “high risk” in two areas, substance abuse and residential instability (see Appendix for risk category ranges), whereas the average rank for other factors and in other groups are within the “moderate risk” range.

### Regression Analyses Results: Differences in Risk by Diagnostic Status

To contextualize our results in prior research, which has not considered differences between those with and without co-occurring SUD, we regressed each risk factor on diagnostic status (i.e., comparing those with and without SPD; see Table 2). After adjusting for demographic differences, people with SPD (with and without SUD) had statistically significant greater risk in all but three domains (history of non-compliance, family criminality, and criminal associates). Prior to taking into account co-occurring SUD (as we do below), people with SPD were greater in risk than those without SPD in the areas of social environment, criminal involvement, substance abuse, residential instability, social isolation, vocational/education, criminal attitudes, financial, leisure and recreation, criminal personality, and history of violence.

**Table 2:** Linear regression results conveying the relationships between diagnostic status and recidivism risk factors.

**Scale (dependent variables)**	**SPD (independent variable)**		
	**β (SE)**	**95% CI**	***p***
Social environment	0.48 (0.14)	[0.21, 0.75]	0.01
Criminal involvement	0.38 (0.12)	[0.13, 0.60]	0.01
History of non-compliance	0.21 (0.13)	[−0.04, 0.46]	0.30
Substance abuse	0.71 (0.14)	[0.45, 0.98]	<0.01
Residential instability	1.33 (0.14)	[1.07, 1.60]	<0.01
Social isolation	1.48 (0.14)	[1.22, 1.76]	<0.01
Vocational/education	1.24 (0.13)	[0.97, 1.50]	<0.01
Criminal attitudes	0.82 (0.14)	[0.55, 1.09]	<0.01
Financial	0.37 (0.14)	[0.09, 0.64]	0.03
Family criminality	0.04 (0.13)	[−0.22, 0.3]	0.77
Leisure and recreation	1.17 (0.14)	[0.90, 1.45]	<0.01
Criminal personality	0.86 (0.14)	[0.58, 1.13]	<0.01
Criminal associates/peers	0.13 (0.14)	[−0.14, 0.40]	0.70
History of violence	0.74 (0.13)	[0.48, 1.00]	<0.01

*Results are based on 14 ordinary least squares regressions comparing risk between those with SPD (serious psychiatric disorder) and without SPD (reference category). Each regression model adjusted for demographic variables (coefficients are omitted). All risk factor scales are measured as deciles. The categories SPD and No SPD include those with and without an SUD. p values are adjusted for multiple comparisons using the Holm method*.

### Regression Analyses Results: Differences in Risk by Diagnostic Group

**Figure 1:**
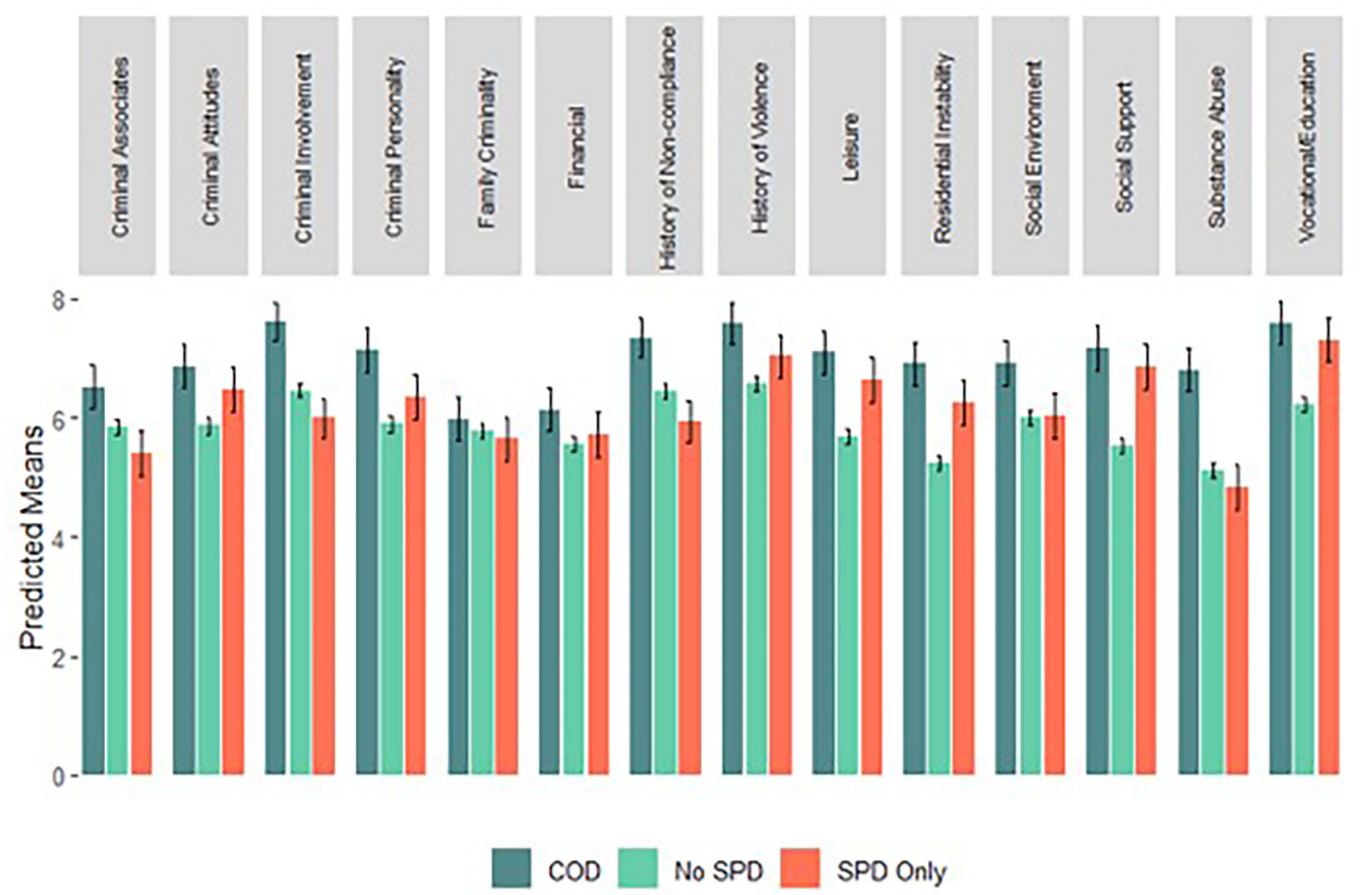
Predicted risk score by diagnostic group. Bars represent predicted mean risk decile scores across diagnostic groups for the typical person on probation (i.e., a 35-year-old, Black man); whiskers represent 95% Confidence Intervals. Predicted means are calculated based on the regression equations presented in Table 3. Categories are coded as mutually exclusive. SPD, serious psychiatric disorder; COD, co-occurring serious psychiatric and substance use disorder. See Appendix for further information on the interpretation of scores.

To answer our research question, we assessed differences in risk between those with no SPD, SPD only, and COD (see Table 3; Figure 1). We found no statistically significant difference between those without SPD and those with SPD only in the domains of criminal associates, criminal personality, family criminality, financial, history of violence, social environment, and substance abuse. Compared to those without SPD, those with SPD only were significantly lower in risk for history of non-compliance and criminal history, averaging about 5 percentile points lower. Though not statistically significant, coefficients were also negative in the areas of criminal associates, family criminality, and substance abuse. Compared to those without SPD, those with SPD only were significantly greater in risk related to criminal attitudes, leisure, residential instability, vocational/education, and social isolation, respectively averaging about 6, 9, 10, 11, and 13 percentile points higher.

**Table 3:** Linear regression results conveying the relationship between diagnostic group and recidivism risk factors (*n* = 4,809).

**Scale (dependent variables)**	**COD**			**SPD Only**		
	**β (SE)**	**95% CI**	***p***	**β (SE)**	**95% CI**	***p***
Social environment	0.91 (0.19)	[0.55, 1.28]	<0.01	0.02 (0.19)	[−0.36, 0.39]	1.00
Criminal involvement	1.15 (0.16)	[0.84, 1.47]	<0.01	−0.46 (0.17)	[−0.79, −0.13]	0.05
History of non-compliance	0.90 (0.17)	[0.56, 1.24]	<0.01	−0.51 (0.18)	[−0.86, −0.17]	0.04
Substance abuse	1.68 (0.18)	[1.32, 2.04]	<0.01	−0.30 (0.19)	[−0.67, 0.07]	0.45
Residential instability	1.66 (0.18)	[1.29, 2.02]	<0.01	1.00 (0.19)	[0.63, 1.37]	<0.01
Social isolation	1.64 (0.19)	[1.28, 2.01]	<0.01	1.32 (0.19)	[0.95, 1.69]	<0.01
Vocational/education	1.38 (0.18)	[1.02, 1.73]	<0.01	1.09 (0.19)	[0.73, 1.46]	<0.01
Criminal attitudes	1.01 (0.19)	[0.64, 1.37]	<0.01	0.63 (0.19)	[0.25, 1.00]	0.01
Financial	0.58 (0.19)	[0.20, 0.95]	0.04	0.15 (0.19)	[−0.23, 0.53]	1.00
Family criminality	0.21 (0.18)	[−0.15, 0.56]	0.26	−0.13 (0.18)	[−0.50, 0.23]	1.00
Leisure and recreation	1.40 (0.19)	[1.03, 1.77]	<0.01	0.94 (0.19)	[0.56, 1.32]	<0.01
Criminal personality	1.24 (0.19)	[0.87, 1.61]	<0.01	0.45 (0.19)	[0.07, 0.83]	0.11
Criminal associates	0.68 (0.19)	[0.31, 1.05]	<0.01	−0.44 (0.19)	[−0.82, −0.07]	0.11
History of violence	1.01 (0.18)	[0.66, 1.36]	<0.01	0.46 (0.18)	[0.11, 0.82]	0.08

*Results are based on 14 ordinary least squares regressions comparing risk between those with SPD only (and no SUD), COD, and no SPD (reference category). Each regression adjusted for demographic variables (coefficients are omitted). All risk factor scales are measured as deciles. SPD, serious psychiatric disorder; COD, co-occurring serious psychiatric and substance use disorder. p values are adjusted for multiple comparisons using the Holm method*.

Assessing differences between participants with no SPD and those with COD, those with COD were at statistically significant greater risk across all domains, except for family criminality. These differences ranged from about 6 to 17 percentile points and, in addition to substance abuse, were starkest for residential instability and social isolation.

### *Post-hoc* Analyses

To assess differences between those with SPD only and with COD, we used Tukey multiple comparison tests. Compared to those with COD, those with SPD only were at lower risk across all domains, with statistically significant differences in criminal associates (*b* = −1.12, *p* < 0.001), criminal history (*b* = −1.61, *p* < 0.001), history of non-compliance (*b* = −1.41, *p* < 0.001), social environment (*b* = −0.90, *p* = 0.012), and substance abuse (*b* = −1.98, *p* < 0.001).

## Discussion

Appropriately assessing risk for recidivism and targeting interventions to reduce that risk require a clear understanding of the factors that relate to criminal involvement for different groups. This need is particularly stark for persons with SPD, who are grossly overrepresented in criminal legal systems. In this study, we assessed the relevance of an array of risk factors for recidivism among a sample of people on probation. We found that, compared to people without SPD, people with SPD (with and without SUD) had significantly greater risk in nearly all risk domains. However, when SUD were taken into account, and those with SPD only were distinguished from those with COD, distinct risk profiles emerged. Overall, results suggest that when considering the risk of recidivism among persons with SPD, it is critical to consider whether said persons have co-occurring SUD. In the remainder of the discussion, we unpack these findings. First, we note study limitations.

This study has three primary limitations. First, we relied on administrative behavioral health data to identify persons with serious psychiatric and substance use disorders. It is possible that we coded persons who have not had contact with this system erroneously as having no SPD. Given the publicly funded behavioral health system is highly accessible in the study site and our rate of SPD is similar to that found in other jail-based samples [e.g., Teplin (11)], we are optimistic that use of administrative data has not substantially biased results in this manner. Second, inclusion criteria may have limited the generalizability of our findings. As noted in Methods, we excluded 27% of people on probation due to missing data, and several demographic, diagnostic, and offense severity differences existed between those included and excluded. In particular, findings are best generalized to groups that have mostly committed felony index offenses. Finally, we relied on data from the COMPAS risk assessment. Though we assessed internal consistency and convergent and discriminant validity for the COMPAS base scales, we did not test predictive validity of base scales. Therefore, though different groups may experience more or less risk in any area, we cannot firmly claim increased recidivism as a result of a preponderance of risk in any base scale. Further, we found the financial scale, measured several related but distinct financial issues and had low internal consistency. When we examined differences in responses by item, we found persons with SPD were more likely than persons without SPD to answer “often” to questions like, “how many times do you have barely enough money to get by?” (45 vs. 34%) but not questions like, “how frequently do you have conflicts with friends/family over money?” (4 vs. 5%). The fact that persons with SPD had significantly higher risk in other scales related to economic status (residential instability and vocational/education), but not the financial scale itself, likely reflects this lack of internal consistency.

With these limitations in mind, we return to study findings. Descriptive data on our sample indicated that variation in risk existed by diagnostic status; persons with SPD (with and without co-occurring SUD) experienced greater risk in all risk areas, except family criminality. Linear models controlling for demographic covariates provide further support for these differences; the presence of SPD (with and without SUD) was significantly associated with greater risk in all but three domains. In other words, without accounting for co-occurring SUD, persons with SPD appear particularly high in risk across a variety of criminal, socioeconomic, and psychological domains. This finding aligns with prior research, which has found that parolees with SPD are relatively high in risk across domains (7).

This study added to existing research by taking COD into account. Given previous research indicating that persons with SPD (with and without COD) have enhanced criminogenic risk, we hypothesized that persons with SPD only and with COD would be relatively high in risk compared to those without SPD. We also hypothesized that, because persons with COD are likely to misuse substances [a consistent criminogenic risk factor (4)] and recidivate more often than other system involved persons (2), people with COD would experience the greatest risk. Contrary to our hypothesis, findings indicated that, relative to persons without SPD, persons with SPD only are at statistically significant increased risk in a minority of domains and are actually at statistically significant reduced risk in two domains (history of non-compliance and criminal history). In support of our hypothesis, we found that people with COD were at particularly high risk. Relative to persons with no SPD, persons with COD are almost unilaterally at increased risk across domains (c.f., family criminality); relative to those with SPD only, persons with COD had statistically significant greater risk in the domains of criminal associates, criminal history, history of non-compliance, social environment, and substance abuse. This suggests that the risk profile set forth in prior research, which suggests that only criminal associates and substance abuse are particularly relevant to persons with SPD (7), likely reflect those with co-occurring disorders but not SPD alone.

Ultimately, we find that the quantity and quality of risk differs by diagnostic group. In practical terms, this means that effective interventions may differ for those with SPD only and those with COD. Compared to those without SPD, persons with SPD are on average ~10–13% higher in risk in the areas of leisure, residential instability, social isolation, and vocational/education. In other words, of the five risk factors disproportionately experienced by persons with SPD, the four greatest are social and economic in nature. Thus, recidivism among persons with SPD may relate disproportionately to these social and economic factors. As such, interventions that enhance economic stability and social connectedness may be particularly relevant for persons with SPD. Meanwhile, system-involved persons with COD, who experience relatively high risk across domains (averaging 10 to 17% higher risk in most), may benefit from interventions that comprehensively address substance use, improve economic circumstances and social support, *and* address other risk factors. This finding provides support for therapeutic community interventions, which are holistic in nature and empirically show promise for reducing recidivism among persons with COD (21).

## Conclusion

Individuals with SPD with and without a substance use problem represent a significant proportion of those incarcerated, on probation, and at a high risk for recidivism. When considering the quantity and quality of risk of recidivism among persons with SPD, this study indicates it is critical to consider whether said persons have co-occurring substance use disorders; persons with COD, on average, are at greater risk of recidivism than their counterparts with SPD only. As such, targeting interventions that broadly focus on dynamic recidivism risk factors, including substance use, are likely to yield positive results in terms of recidivism reduction among persons with COD. As for persons with SPD only, interventions that improve social connectedness and economic circumstances seem particularly warranted.

## Data Availability Statement

The datasets presented in this article are not readily available because the data for this study are not available for pubic dissemination due to constraints established in related data use agreements. Requests to access the datasets should be directed to leahjacobs@pitt.edu.

## Ethics Statement

The studies involving human participants were reviewed and approved by Human Research Protection Office, University of Pittsburgh. Written informed consent for participation was not required for this study in accordance with the national legislation and the institutional requirements.

## Author Contributions

LJ conceptualized and operationalized the study aim and approach, as well as collected, linked, and coded all data. AF and LJ conducted the statistical analyses. LJ, TL, AG, and CN each wrote sections of the manuscript. TL was also consulted in the development of analyses. All authors contributed to the development and revision of the manuscript, and read and approved the submitted version.

## Funding

This research was supported by Award Number T32AA007240 from the National Institute on Alcohol Abuse and Alcoholism. This work was also supported by a Central Research Development Fund award from the University of Pittsburgh Office of the Provost and APC charges for this article were paid by the University Library System, University of Pittsburgh.

## Author Disclaimer

The content is solely the responsibility of the authors and does not necessarily represent the official view of the National Institute on Alcohol Abuse and Alcoholism or the National Institutes of Health.

## Conflict of Interest

The authors declare that the research was conducted in the absence of any commercial or financial relationships that could be construed as a potential conflict of interest.

## Publisher's Note

All claims expressed in this article are solely those of the authors and do not necessarily represent those of their affiliated organizations, or those of the publisher, the editors and the reviewers. Any product that may be evaluated in this article, or claim that may be made by its manufacturer, is not guaranteed or endorsed by the publisher.

## References

[1] Mulvey EP Schubert CA. Mentally Ill individuals in jails and prisons. Crime Just. (2017) 46:231–77. 10.1086/688461

[2] Baillargeon J Penn JV Knight K Harzke AJ Baillargeon G Becker E. Risk of Reincarceration among prisoners with co-occurring severe mental illness and substance use disorders. Adm Policy Ment Health. (2010) 37:367–74. 10.1007/s10488-009-0252-9 19847638

[3] Monahan J Skeem J Lowenkamp C. Age, risk assessment, and sanctioning: overestimating the old, underestimating the young. Law Hum Behav. (2017) 41:191–201. 10.1037/lhb0000233 28150973

[4] Bonta J Andrews DA. The Psychology of Criminal Conduct. New York, NY: Routledge (2016). p. 470.

[5] Andrews DA Bonta JB. Rehabilitating criminal justice policy and practice. Psychol Public Policy Law. (2010) 16:39–55. 10.1037/a0018362

[6] Bonta J Blais J Wilson HAA. theoretically informed meta-analysis of the risk for general and violent recidivism for mentally disordered offenders. Aggress Violent Behav. (2014) 19:278–87. 10.1016/j.avb.2014.04.014

[7] Skeem JL Winter E Kennealy PJ Louden JE Tatar II JR. Offenders with mental illness have criminogenic needs, too: toward recidivism reduction. Law Hum Behav. (2014) 38:212–24. 10.1037/lhb0000054 24377913

[8] Morgan RD Fisher WH Duan N Mandracchia JT Murray D. Prevalence of criminal thinking among state prison inmates with serious mental illness. Law Hum Behav. (2010) 34:324–36. 10.1007/s10979-009-9182-z 19551496 PMC2987583

[9] Wilson AB Farkas K Ishler KJ Gearhart M Morgan R Ashe M. Criminal thinking styles among people with serious mental illness in jail. Law Hum Behav. (2014) 38:592–601. 10.1037/lhb0000084 24707911

[10] Abram KM Teplin LA McClelland GM. Comorbidity of severe psychiatric disorders and substance use disorders among women in jail. Am J Psychiatry. (2003) 160:1007–10. 10.1176/appi.ajp.160.5.1007 12727711

[11] Teplin LA. Psychiatric and substance abuse disorders among male urban jail detainees. Am J Public Health. (1994) 84:290–3. 10.2105/AJPH.84.2.290 8296957 PMC1614991

[12] James DJ, Glaze, LE,. Mental health problems of prison jail inmates. Washington, DC: Bureau of Justice Statistics (US). (2006). Available online at: https://bjs.ojp.gov/library/publications/mental-health-problems-prison-and-jail-inmates

[13] McNiel DE Binder RL Robinson JC. Incarceration associated with homelessness, mental disorder, and co-occurring substance abuse. Psychiatr Serv. (2005) 56:840–6. 10.1176/appi.ps.56.7.840 16020817

[14] Lipari RN, Park-Lee, E,. Key substance use mental health indicators in the United States: Results from the 2018 national survey on drug use health. Rockville, MD: SAMHSA. (2019). Available online at: https://www.samhsa.gov/data/sites/default/files/cbhsq-reports/NSDUHNationalFindingsReport2018/NSDUHNationalFindingsReport2018.pdf

[15] Demarais SL Johnson KL. Singh JP. Performance of recidivism risk assessment instruments in US correctional settings. Psychol Serv. (2016) 13:206–22. 10.1037/ser0000075 27267819

[16] Brennan T Dieterich W Ehret B. Evaluating the predictive validity of the COMPAS risk and needs assessment system. Crim Justice Behav. (2009) 36:21–40. 10.1177/0093854808326545

[17] Jacobs LA Skeem JL. Neighborhood risk factors for recidivism: for whom do they matter? Am J Community Psychol. (2021) 67:103–15. 10.1002/ajcp.12463 32960992 PMC7969362

[18] American Psychiatric Association. Diagnostic and Statistical Manual of Mental Disorders (4th ed. revised). Washington, D.C.: American Psychiatric Association (2000).

[19] American Psychiatric Association. Diagnostic and Statistical Manual of Mental disorders (5th ed.). Washington, D.C.: American Psychiatric Association (2013). 10.1176/appi.books.9780890425596

[20] R Core Team. R: A language and environment for statistical computing. R Foundation for Statistical Computing (2020).

[21] Perry AE Martyn-St James M Burns L Hewitt C Glanville JM Aboaja A. Interventions for drug-using offenders with co-occurring mental health problems. Cochr Database Systematic Rev. (2019) 10:CD010901. 10.1002/14651858.CD010901.pub3 31588993 PMC6778977

