# Supplementary material for: Risk Factors for Criminal Recidivism Among Persons With Serious Psychiatric Diagnoses: Disentangling What Matters for Whom
Source: Front Psychiatry. 2021 Dec 16;12:778399. doi: 10.3389/fpsyt.2021.778399 (PMC8716805; doi:10.3389/fpsyt.2021.778399)
Supplement: Supplementary file 1 [file Data_Sheet_1.docx]

| **Appendix A.**  *Description of COMPAS core scales* | | | |
| --- | --- | --- | --- |
| Scale | Description | Interpretation of decile scores | Cronbach’s alpha |
| Criminal involvement | Four item scale measuring the magnitude of the person’s involvement with the criminal legal system, as determined by counts of the number of times they have been arrested, charged, incarcerated, and sentenced. | Low risk (1 – 4)  Moderate risk (5 – 7)  High risk (8 – 10) | α = .80 |
| History of non-compliance | Five item scale that assesses a person’s lack of compliance with previous sentences, such as violations of probation, instances of recidivism, and revocation of community supervision status. | Low risk (1 – 4)  Moderate risk (5 – 7)  High risk (8 – 10) | α = .80 |
| History of violence | Nine item scale that considers the severity and amount of violence in a person’s background, primarily measured by the number and types of previous violent offenses and arrests. | Low risk (1 – 4)  Moderate risk (5 – 7)  High risk (8 – 10) | α = .63 |
| Social environment | Six item scale that measures how much exposure to crime a person may have in their neighborhood. Items assess the person’s neighbors or family members’ likelihood of being victimized or carrying weapons, and perceived prevalence of drugs and gangs in the neighborhood. | Low risk (1 – 5)  Moderate risk (6 – 7)  High risk (8 – 10) | α = .95 |
| Residential instability | Ten item scale that evaluates a person’s housing stability and their ties to their community. Questions assess housing status, length of time in the residence and neighborhood, and with whom the person lives. | Low risk (1 – 5)  Moderate risk (6 – 7)  High risk (8 – 10) | α = .79 |
| Social isolation | Eight item scale that determines the extent to which a person has access to social support and close relationships. Items assess a person’s feelings of loneliness, feelings of social belonging and inclusion, and whether the person has close relationships with people who know them well and who they can rely on for emotional support. | Low risk (1 – 5)  Moderate risk (6 – 7)  High risk (8 – 10) | α = .82 |
| Criminal associates/peers | Seven item scale that reflects the degree to which a person’s social circle exposes them to criminal opportunities, such as whether they are or have been in a gang, and the number of friends who are involved in the criminal legal system in some way, are gang members, or regularly use drugs. | Low risk (1 – 4)  Moderate risk (5 – 7)  High risk (8 – 10) | α = .75 |
| Family criminality | Eight item scale that assesses how much exposure to the criminal legal system a person might have had by way of the involvement of kin. Items measure whether family members have been arrested, used drugs or spent time incarcerated. | Low risk (1 – 5)  Moderate risk (6 – 7)  High risk (8 – 10) | α = .83 |
| Vocational/education | Twelve item scale that measures the extent to which a person has successfully engaged with vocational or educational institutions, as measured by indicators such as the amount of time they spent in school, grades, disciplinary history, and the completion of degrees; and their work skills, expectations for pay, their desire for further training, and their expectations about their ability to succeed at work. | Low risk (1 – 5)  Moderate risk (6 – 7)  High risk (8 – 10) | α = .70 |
| Financial | Five item scale comprised of items that assess whether the person struggles with their finances, has access to well-paying jobs, or has regular social conflicts related to money. | Low risk (1 – 5)  Moderate risk (6 – 7)  High risk (8 – 10) | α = .64 |
| Criminal attitudes | Ten item scale that measures a person’s perceptions about crime, and specifically whether they hold beliefs that provide moral justifications for illegal behaviors or are inclined toward violence. | Low risk (1 – 5)  Moderate risk (6 – 7)  High risk (8 – 10) | α = .81 |
| Criminal personality | Thirteen item scale that measures a person’s tendency toward manipulative, self-centered, or aggressive thinking or behaviors. | Low risk (1 – 5)  Moderate risk (6 – 7)  High risk (8 – 10) | α = .70 |
| Substance abuse | Ten item scale that provides a general assessment of problematic substance use as measured by the perceived influence of drugs and/or alcohol on current or previous criminal actions, history with drug and alcohol treatment, or desire for drug and alcohol treatment. | Low risk (1-2)  Medium risk (3-4)  High risk (5-10) | α = .89 |
| Leisure and recreation | Five item scale that assesses how often a person is bored or restless and whether they feel they have interesting things to do. | Low risk (1 – 5)  Moderate risk (6 – 7)  High risk (8 – 10) | α = .82 |
| *Note.* Descriptions are summaries of items from 14 COMPAS core scales, as used by the study site. COMPAS is a proprietary instrument owned by Equivant. Decile score interpretation is drawn from Northpointe (1). For further detail on scales and items, see Northpointe (2). | | | |

1. Northpointe Institute for Public Management, Inc. Measurement and Treatment Implications of COMPAS Core Scales. (2009). https://www.michigan.gov/documents/corrections/Timothy_Brenne_Ph.D.__Meaning_and_Treatment_Implications_of_COMPA_Core_Scales_297495_7.pdf

(2) Northpointe Institute for Public Management, Inc. COMPAS Scales and Risk Models Validity and Reliability. (2010). https://archive.epic.org/algorithmic-transparency/crim-justice/EPIC-16-06-23-WI-FOIA-201600805-COMPASSummaryResults.pdf
